# Ultrafast Ultrasound Imaging for Super-Resolution Preclinical Cardiac PET

**DOI:** 10.1007/s11307-020-01512-w

**Published:** 2020-06-29

**Authors:** Mailyn Perez-Liva, Thulaciga Yoganathan, Joaquin L. Herraiz, Jonathan Porée, Mickael Tanter, Daniel Balvay, Thomas Viel, Anikitos Garofalakis, Jean Provost, Bertrand Tavitian

**Affiliations:** 1 Université de Paris, PARCC, INSERM, 56, rue Leblanc, 75015 Paris, France; 2 Nuclear Physics Group and IPARCOS, Complutense University of Madrid, Plaza de las Ciencias, 1, 28020, Madrid, Spain; 3 Health Research Institute of the Hospital Clínico San Carlos (IdISSC), Madrid, Spain; 4 Physics for Medicine Paris, Inserm/ESPCI Paris-PSL/PSL-University/CNRS, 17 rue Moreau, 75012, Paris, France; 5 Engineering physics department, Polytechnique Montréal, Montréal, Canada; 6 Montreal Heart Institute, Montréal, Canada; 7 Service de Radiologie, APHP Centre, Hôpital Européen Georges Pompidou, Paris, France

**Keywords:** Cardiac Positron Emission Tomography, Super-resolution, Ultrafast Ultrasound Imaging, Image quality

## Abstract

**Purpose:**

Physiological motion and partial volume effect (PVE) significantly degrade the quality of cardiac positron emission tomography (PET) images in the fast-beating hearts of rodents. Several Super-resolution (SR) techniques using *a priori* anatomical information have been proposed to correct motion and PVE in PET images. Ultrasound is ideally suited to capture real-time high-resolution cine images of rodent hearts. Here, we evaluated an ultrasound-based SR method using simultaneously acquired and co-registered PET-CT-Ultrafast Ultrasound Imaging (UUI) of the beating heart in closed-chest rodents.

**Procedures:**

The method was tested with numerical and animal data (*n* = 2) acquired with the non-invasive hybrid imaging system PETRUS that acquires simultaneously PET, CT, and UUI.

**Results:**

We showed that ultrasound-based SR drastically enhances the quality of PET images of the beating rodent heart. For the simulations, the deviations between expected and mean reconstructed values were 2 % after applying SR. For the experimental data, when using Ultrasound-based SR correction, contrast was improved by a factor of two, signal-to-noise ratio by 11 %, and spatial resolution by 56 % (~ 0.88 mm) with respect to static PET. As a consequence, the metabolic defect following an acute cardiac ischemia was delineated with much higher anatomical precision.

**Conclusions:**

Our results provided a proof-of-concept that image quality of cardiac PET in fast-beating rodent hearts can be significantly improved by ultrasound-based SR, a portable low-cost technique. Improved PET imaging of the rodent heart may allow new explorations of physiological and pathological situations related with cardiac metabolism.

**Electronic supplementary material:**

The online version of this article (10.1007/s11307-020-01512-w) contains supplementary material, which is available to authorized users.

## Introduction

Standing as a paradox with respect to the vast number of applications in oncology and neurology, cardiac metabolic imaging using 2-deoxy-2-[^18^F]fluoro-D-glucose (FDG) positron emission tomography (PET) has so far found few clinical applications. One reason is the difficulty to attribute changes in FDG cardiac uptake to pathological *versus* physiological causes, *e.g.* substrate availability, energetic demand, and neural and hormonal regulation [1, 2]. Under basal conditions, the energetic metabolism of the normal adult heart is mostly supported by the oxidation of fatty acids. Other substrates such as lactate, ketone bodies, and glucose contribute minorly [2]. However, in conditions of increased cardiac energy demand, both physiological, *e.g.* strenuous exercise, and pathophysiological, *e.g.* acute ischemia, myocarditis, left ventricle hypertrophy, systemic hypertension, and drug toxicity, there is a switch towards glycolysis and, just as in the fetal heart, glucose becomes the preferred source of fuel of the adult heart [3–6]. PET imaging of the heart in rodent models would facilitate the exploration of the connection between the level of glucose metabolism and cardiac (patho)physiology. However, high-quality imaging of the rodent heart remains a technical challenge because of the gap between the dimensions of the heart’s movements, *i.e.* left ventricle displacement-velocity of around 2–3 cm/s [7–10] at 300–800 beats per minute [11], and the spatial and temporal resolutions of preclinical PET scanners, over 1 mm [12] and seconds to minutes, respectively [7]. Therefore, each cardiac PET image accumulates several heart cycles, leading to significant blurring, motion-induced artifacts, and imprecise radioactivity quantification. Gating raw PET data along the cardiac cycles synchronously with an electrocardiogram (ECG) compensates for cardiac motion (Gated Cardiac-PET [13]), but comes at the cost of lower counting statistics and contrast.

A number of methods, collectively termed super-resolution (SR), have been proposed to improve the spatial resolution of PET [14–20]. SR is based on iterative inverse algorithms that use *a priori* information from an anatomical reference in order to (i) characterize the motion vector fields (MVF) of the heart’s motion and (ii) increase spatial sampling through shifts with sub-resolution precision [15, 21, 22]. Gated Cardiac-PET frames are readily available priors for SR, but they are far from ideal due to their high noise content and low resolution [15, 22], and most SR methods use a second imaging modality, such as X-ray tomography (CT) or magnetic resonance imaging (MRI). Ultrasound is the routine reference technique for real-time, anatomical, cardiac imaging in humans and rodents, with excellent temporal (≤ 1 ms) and spatial (~ 0.1 mm) resolution, high portability, and relatively low-cost. However, to the best of our knowledge, it has never been used as prior for SR correction of cardiac PET.

Here we assessed an ultrasound-based SR technique in rodent hearts using the non-invasive *in vivo* preclinical imaging instrument PETRUS [9, 23] that merges simultaneously acquired and co-registered PET-CT and Ultrafast Ultrasound Imaging (UUI) [24]. Cardiac PETRUS allows the acquisition, during the same imaging session, of glucose metabolism and real-time high resolution cardiac motion with negligible effects on PET image quality [9]. This ultrasound-based SR method was applied in the image domain, and its performance was tested on simulated and real preclinical datasets.

## Materials and Methods

### Super-resolution in the Image Domain

SR restoration is an ill-conditioned problem since it aims to restore a high-resolution version of an object from a set of low-resolution images of it, which are related through a series of convolutional degrading steps such as motion, blurring, down-sampling, and noise corruption:

1 $$ {L}_i={BDM}_iH+{\Psi}_i,\kern21em $$

being *H* a high-resolution image of the inspected object, *L*_*i*_ a set containing *i* = 1, …, *N*_*f*_ low-resolution image frames of the object, *N*_*f*_ is the total number of frames, *M*_*i*_ the MVF containing the spatio-temporal geometrical transformation of the object motion at frame *i*, *B* a blurring kernel or point spread function (PSF) of the imaging system, *D* a down-sampling kernel defining the difference in pixel size between *H* and *L*_*i*_*, i.e. D* combines several voxels of the grid into one, for example using linear decimation, and Ψ_*i*_ is an additive noise term characterized as a gaussian random variable with zero mean and variance σ^2^.

Let us define Γ_*i*_ : Ω → *ℝ* as a dataset containing low-resolution frames of a Gated Cardiac-PET, where Ω defines de image domain. Using Γ_*i*_, a high-resolution original image is estimated as in [14], using a Maximum *a Posteriori* (MAP) variational approach [25] to minimize the functional $F=\frac{1}{2}\sum \limits_i{\left|{\Gamma}_i-{\mathrm{L}}_i\right|}^2$:

2 $$ {\hat{H}}_{\mathrm{MAP}}=\underset{H\in {\mathbb{R}}^{\Omega}}{\mathrm{argmin}}\frac{1}{2}\sum \limits_i{\left|{\Gamma}_i-{L}_i\right|}^2+\lambda TV(H),\kern12.25em $$

where ${\hat{H}}_{\mathrm{MAP}}$ is the optimal high-resolution image that solves the variational problem of Eq. (2), *TV*(*H*) is a penalizing function that measures the quality of the restored image by countering the effect of the noise term Ψ_*i*_, and *λ* balances the weight of the penalization term. Regarding the uptake FDG in the cardiac ventricular wall, we expect a clear difference between healthy and injured tissue but very little variation of uptake inside the healthy tissue. Hence, we adopted a Total Variation (TV) model [14] as penalizing term because it is suitable for restoring piece-wise structures and to preserve edges:

3 $$ TV(H)=\sum \limits_{x\in \Omega}\mid \nabla H(x)\mid dx.\kern7em $$

In Eq. (3), ∇ denotes the gradient operator and *x* is the position in the image domain. To solve the Euler-Lagrange equation associated to the variational problem of Eq. (2), we use a steepest-descent algorithm:

$$ {H}^{n+1}={H}^n+{\nabla}_H\mathrm{F}+\kern0.5em \lambda \nabla \frac{\nabla {H}^n}{\mid \nabla {H}^n\mid }, $$

4 $$ {\nabla}_H\mathrm{F}=\frac{1}{N_f}{\sum}_{i=1}^{N_f}{B}^{-1}{M_i}^{-1}{D}^{-1}\left({\Gamma}_i-{L^n}_i\right),\kern17.75em $$

where *L*^*n*^_*i*_ is a simulated set of low-resolution images that can be obtained through Eq. (1) using the high-resolution image *H*^*n*^, where *n* = 1, …, *N*, defines the iterations of the algorithm, *D*^−1^ is the inverse of the down-sampling operator that performs the up-sampling of data, *i.e.* it divides each voxel of the grid in smaller voxels. *M*_*i*_^−1^ inverts the MVF (we use the approximation *M*_*i*_^−1^ =  − *M*_*i*_), and the deblurring kernel *B*^−1^ is the inverse of the blurring kernel *B*.

### Ultrasound-Based Motion Registration Algorithm

The motion estimation of the myocardial during a cardiac cycle consists in performing a sequence of image-to-image registration problems between each frame at time interval *i* and the initial frame *i* = 1. We used UUI-B-mode in order to characterize the MVF, *i.e.* the *M*_*i*_ term in Eq. (1). We employed a variational approach to perform the registration of each frame by minimizing an energy functional *E* defined in a spatial window Υ {*x*_*j*_|*j ϵ* Υ} around the left ventricle. To account for changes of intensity that might appear in cardiac ultrasonic sequences, such as ribs’ absorption and speckle noise, we combined local and global image estimators by characterizing the local phase and intensity of the images [22]:

5 $$ E=\frac{1}{2}\sum \limits_{j\ \epsilon\ \mathrm{Y}}{\int}_{\Omega}\delta \left(x-{x}_j\right)\left\{k{\left[{I}_R\left(\mathrm{x}\right)-{I_T}_i\left(x+M(x)\right)\right]}^2+\left(1-k\right){\left[{\theta}_R\left(\mathrm{x}\right)-{\theta_T}_i\left(x+M(x)\right)\right]}^2\right\} dx, $$

where *I*_*Ti*_ and *I*_*R*_ are the image template (at frame *i*) and reference, respectively, and *θ*_*Ti*_ and *θ*_*R*_ are the local phases of the image template and reference, respectively. *δ* is the Dirac function, and *k* is a weighting factor to balance the contribution of the intensity and local phase terms in the registration (*k* ≤ 1). We adopted a fluid-like regularization, which can be approximated by a Gaussian filter [26], and it was applied assuming that *M* is the result of the convolution of an auxiliary field *m* with a gaussian kernel *w*_*s*_ of scale *s*:

6 $$ M(x)=\left[{w}_s\ast m\right](x)={\int}_{\varOmega }{w}_s\left(x-{x}^{\prime}\right)m\left({x}^{\prime}\right)d{x}^{\prime }.\kern3em $$

We used a gradient descent method to minimize the functional *E* with respect to *M*:

7 $$ {M}_{i+1}={M}_i-{\nabla}_ME={M}_i-{w}_s\ast \left[{\sum}_{j\ \epsilon\ \mathrm{Y}}\delta \left(x-{x}_j\right){\nabla}_mE\right] $$

As is often used in Demon-like registrations [27, 28], to gain stability around small values of ∇*I*_*R*_ and ∇*θ*_*R*_, we employed a regularized version of the gradient ∇_*m*_*E* as in [27, 28]:

8 $$ {M}_{i+1}={M}_i-{w}_s\ast \sum \limits_{j\ \epsilon\ \mathrm{Y}}\delta \left(x-{x}_j\right)\left[\frac{k\left({I}_R-{I_T}_i\right)\nabla {I}_R}{{\left\Vert \nabla {I}_R\right\Vert}^2+{\left({I}_R-{I_T}_i\right)}^2}+\kern0.5em \frac{\left(1-\mathrm{k}\right)\left({\theta}_R-{\theta_T}_i\right)\nabla {\theta}_R}{{\left\Vert \nabla {\theta}_R\right\Vert}^2+{\left({\theta}_R-{\theta_T}_i\right)}^2}\right].\kern3.5em $$

The local phase *θ*(*x*), which does not depend on the intensity information but on the extraction of local features, *e.g.* points, edges, *etc.*, can be obtained from the monogenic signal using quadrature filters [29, 30]. These filters are the combination of an even-symmetric band-pass filter (*F*_*e*_, giving as result *I*_*e*_ = *F*_*e*_ ∗ *I*, the even component of an image *I*) and of two consecutive odd-symmetric filters (*F*_*o*1_ and *F*_*o*2_) applied to the even component of the signal:

9 $$ \theta ={\mathit{\tan}}^{-1}\left(\frac{I_e}{\sqrt{{\left({F}_{o1}\ast {I}_e\right)}^2+{\left({F}_{o2}\ast {I}_e\right)}^2}}\right). $$

We employ a log-Garbor radial filter as even filter and its Riesz transform as odd filters as in [31]. In order to estimate large motions, a multi-scale pyramidal refinement approach was adopted using 3 decreasing pixel size scales. Consequently, we used three center frequency for the band-pass of the log-Garbor filter as 16, 8, and 2 pixels, for each consecutive decreasing scales. At each scale, we perform 10 iterations and used additive corrections. For the fluid-like regularization (term *w*_*s*_ in Eq. (6)), we used a Gaussian filter with a fixed scale of 10 pixels.

### Implementation Details for the Ultrasound-Based SR of Cardiac PET

**Fig. 1:**
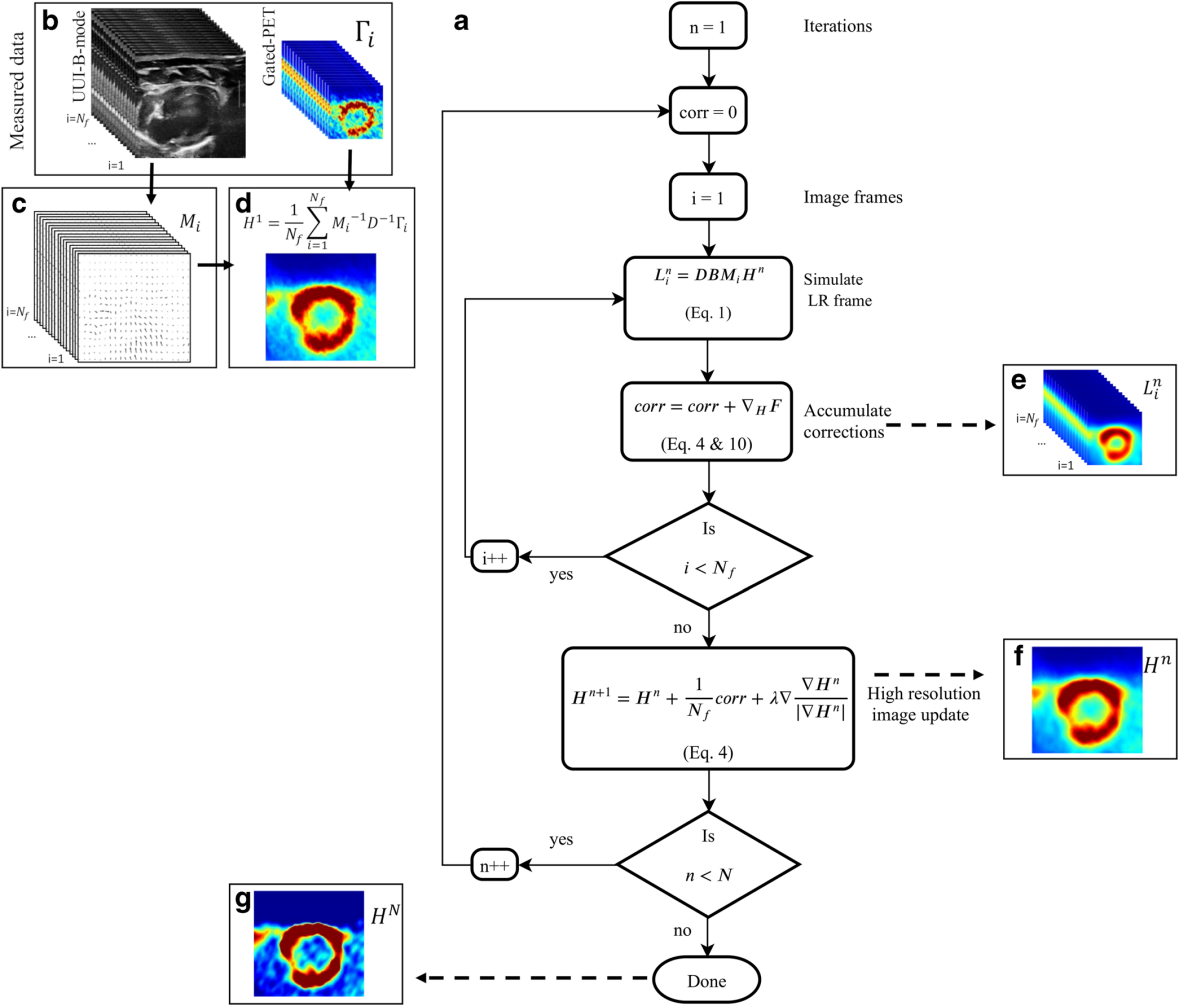
**a** Schematic flow chart of the ultrasound-based-SR method to correct cardiac PET. **b** Multimodal PETRUS data simultaneously acquired and co-registered. **c** UUI-B-mode estimated motion vector fields of the heart’s deformation. **d** The initial high-resolution (HR) guess image used in the ultrasound-based SR is the up-sampling of the result of registering and averaging all the low-resolution (LR) gated PET frames. **e** Using Eq. (1), the HR image simulates LR frames. **f** An averaged correction for the current HR image is calculated using Eq. (4) and regularized using TV, providing the HR image obtained at iterative step *n* of the algorithm. **g** Final ultrasound-based SR image.

Figure 1 a presents the flow chart of the complete ultrasound-based SR restoration problem for cardiac-PET. The algorithm is feed with the Γ_*i*_ dataset of *N*_*f*_ low-resolution Gated Cardiac-PET images and the co-registered UUI-B-mode *N*_*f*_ frames (Fig. 1b). The UUI-B-mode set, which have a smaller pixel size and a better spatial resolution than the PET, is used to estimate the geometrical transformation *M*_*i*_ between frames (Fig. 1c). The algorithm starts with an initial high-resolution guess *H*^1^, which is the image resulting from warping and averaging the *N*_*f*_ Gated Cardiac-PET, up-sampled to the dimensions of the UUI-B-mode maps (Fig. 1d). As in Eq. (1), *H*^1^ is then (i) warped using each *M*_*i*_, (ii) down-sampled to the dimensions of Γi, and (iii) blurred to produce *L*_*i*_^*n*^(Fig. 1e). The blurring kernel is the estimated PSF of the PET scanner of PETRUS, which we previously characterized [9] as a Gaussian kernel with a FWHM of 1.5 mm at the center of the field-of-view. The deblurring kernel *B*^−1^should ideally be the inverse of the blurring kernel *B*. However, as the PSF of a PET scanner is generally represented as a Gaussian kernel, its exact inverse would lead to an ill-posed problem. Hence, we used a simple delta-function with a width of one pixel. Several studies have shown that such simplified backward operators produce correctly reconstructed images [32–34]. This simplifies the term ∇_*H*_F in Eq. (4) as follows:

10 $$ \kern0.5em {\nabla}_H\mathrm{F}=\frac{1}{N_f}{\sum}_{i=1}^{N_f}{M_i}^{-1}{D}^{-1}\left({\Gamma}_i-{L^n}_i\right) $$

For all frames *i*, each difference Γ_*i*_ − *L*^*n*^_*i*_ is up-sampled, warped-back to the selected frame of reference and averaged for all *N*_*f*_. Using the current *n* estimation of the high-resolution image *H*^*n*^, the divergence of the gradient of the TV term (last term in Eq. (4)) is calculated and the *n + 1* estimation of the high-resolution image is obtained using Eq. (4). To evaluate the gradient, border elements of the image are padded by replication outside the boundaries of the image. The parameters λ and k were empirically set to 1 × 10^−6^ and 0.5 respectively, as they provided, respectively, a satisfying tradeoff between noise control and spatial resolution in our experiments (see “Numerical Experiments” for details about the definitions of noise and spatial resolution used), and a consisting motion registration (see supplementary material). All these steps are reiterated until the root-mean-square-error (RMSE) between estimated and measured low-resolution images varies by less than 5 % in two consecutive iterations.

### Numerical Experiments

The performance of the ultrasound-based SR algorithm was tested on a numerical phantom simulating a realistic gated cardiac-PET acquisition in a numerical rat, using the ROBY phantom [12]. Assuming that respiratory and cardiac motions were perfectly cyclic, we simulated 8 respiratory frames of 0.09 s duration each, over a period of 0.72 s, which was the total length of the respiratory cycle and total length of the simulation performed, *i.e.* we simulated 1 respiratory cycle. For the respiratory motion, we choose 1-mm of maximum diaphragm displacement. Each simulated cardiac cycle had a duration of 0.09 s with 8 cardiac frames per respiratory frame. Input simulation images consisted in 256 × 256 × 237 voxels of 0.4 × 0.4 × 0.4 mm covering the thoracic cage of the ROBY phantom. The phantom was simulated using the Monte Carlo software MCGPU-PET [35], a fast simulator which takes into account the main relevant physical processes of the emission, transport, and detection of the radiation. The MCGPU-PET code is an adaptation of the MC-GPU software developed for X-ray imaging [36]. The libraries of cross-sections have been adapted to the 511 keV annihilation gamma rays. Two new modules have been written for the PET modality of the MC-GPU software: the photon source module that models the activity of each voxel, and the phase space detector module that tallies the location and arrival time of the photons on a cylinder around the object. MCGPU-PET allows the use of voxelized PET and CT images as input and provides different output formats including 3D-sinograms of the true and the scatter coincidences. The code selects the coincidences taking into account an energy resolution and energy window, but without including the effects of the transport of the gamma rays in the detector (*i.e.* it assumes a perfect detection). We used a generic preclinical scanner model, similar to the Inveon PET preclinical scanner [37], with an associated spatial resolution at the center of the scanner of ~ 1.5 mm. Each simulation was based on the specific distribution of the activity and materials of each particular frame and contained around 65 million counts, including trues and scatter coincidences. The acquired data of each frame were stored in 527 sinograms (direct and oblique), containing 129 and 168 radial and angular bins, respectively. The simulated data were reconstructed with GFIRST [38], using a 3D-OSEM algorithm and including attenuation and scatter corrections. These corrections were obtained from the known material distribution in each frame, using a 2-tissues class segmentation (air and tissue). The images were reconstructed using 128 × 128 × 127 voxels of 0.8 × 0.8 × 0.746 mm. The whole simulation and reconstructions took in total ~ 52 min with a single GPU (GeForce GTX 1080, 1.73GHz, 8Gb).

We regionally limited the SR analysis to a single 2D slice in transversal orientation. We used the phantom activity information as anatomical reference to estimate the MVF, a down-sampling factor *D* of one every two pixels, and a Gaussian filter with FWHM of 1.5 mm as blurring kernel *B*. Mean pixels’ values of regions of interest (ROIs) located in the left ventricle wall and in the ventricle’s cavity were quantified. In order to simulate a static PET acquisition, we averaged the respiratory and cardiac frames of the reconstructed PET images. To simulate a gated cardiac-PET sequence, all the respiratory frames were averaged within each phase of the cardiac cycle. As the observed effect of respiration in the simulated gated cardiac-PET was a small rigid-translation in the craniocaudal direction, a rigid registration was performed between the first frame of the reference anatomical image and the first frame of the simulated gated cardiac-PET before the registration of the cardiac phases. The estimated registration transformation was then applied to each frame of the gated cardiac-PET.

The improvement in image quality by the SR processing was assessed by measuring the signal-to-noise ratio (SNR), contrast and spatial resolution of the images. The SNR was calculated using the standard deviation (STD) and mean value (MEAN) in the ROIs:

11 $$ \mathrm{SNR}\ (dB)=10\log \left(\frac{\mathrm{MEAN}}{\mathrm{STD}}\right).\kern17em $$

Contrast was defined as Weber’s fraction [39] using the mean value in the left ventricle wall ROI (MEAN_wall_) and in the cavity of the ventricle ROI (MEAN_cav_).

12 $$ \mathrm{Contrast}=\frac{\mathrm{MEA}{\mathrm{N}}_{\mathrm{wall}}-\mathrm{MEA}{\mathrm{N}}_{\mathrm{cav}}}{\mathrm{MEA}{\mathrm{N}}_{\mathrm{cav}}}.\kern17.25em $$

Spatial resolution was defined as the lateral spread function (LSF) of the ventricle’s wall. This was evaluated as the FWHM of a Gaussian function fitted to the mirrored duplicated points, external to the edge of the ventricle’ wall, in a profile crossing the wall. The location of the external edge of the wall was extracted from the matching anatomical reference profile. The procedure was repeated on 5 intensity profiles drawn orthogonally to the heart wall, clockwise: basal lateral, mid lateral, apical, mid-septal, and basal septal [40]. Resolution was then defined as the average of the five estimated LSF.

### PETRUS: Positron Emission Tomography Registered Ultrasonography

**Fig. 2:**
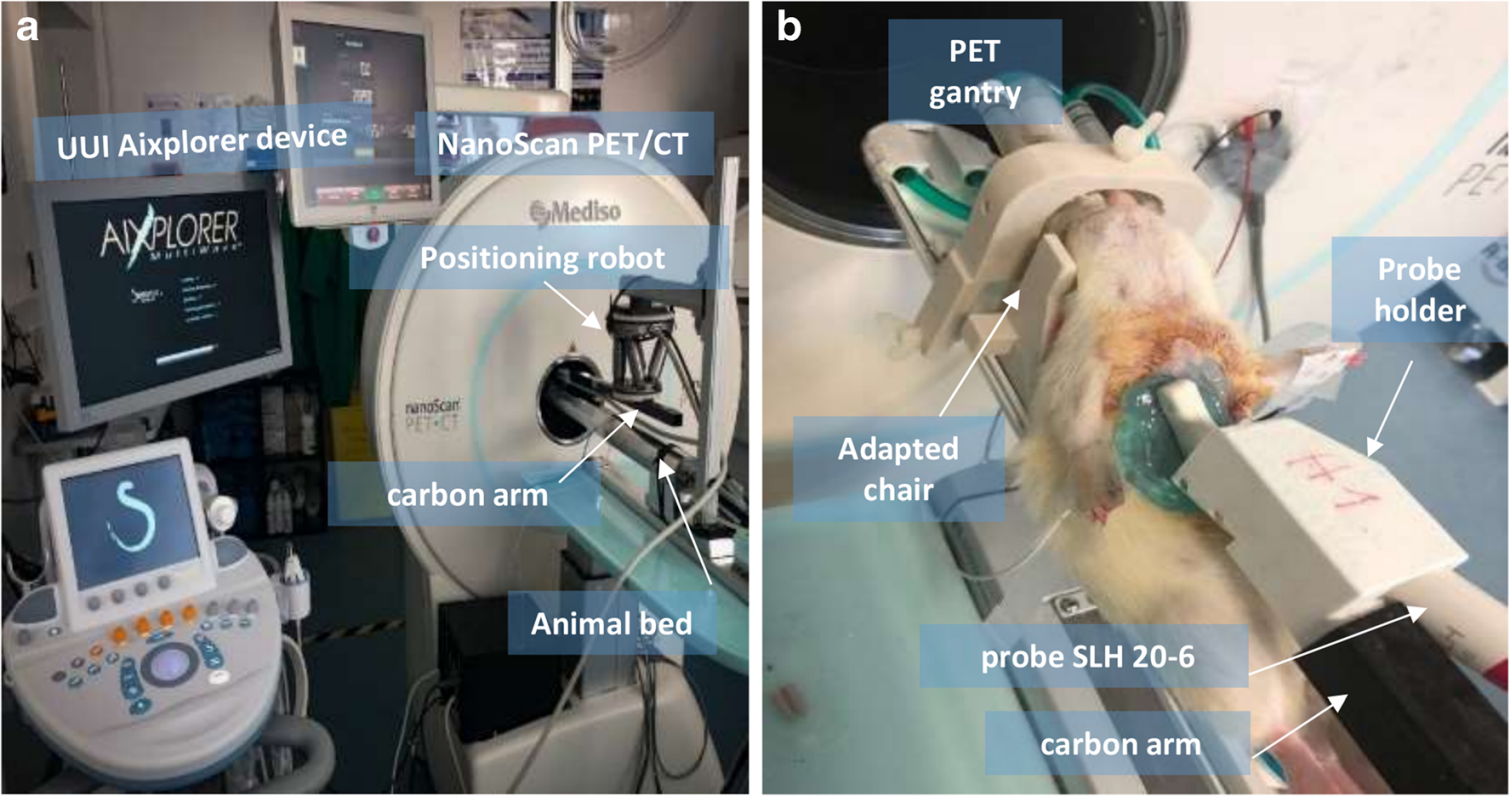
PETRUS setup for cardiac exploration in rodents. **a** Elements of the PETRUS system. **b** Animal location for PETRUS cardiac studies in rats.

PETRUS (Fig. 2) has been described in detail in [23]. Briefly, it combines a preclinical PET-CT scanner for small animals (nanoScan Mediso Ltd., Hungary) with a clinical UUI scanner (Aixplorer, Supersonic Imagine France). The UUI component of PETRUS provides thousands of images per second, allowing the exploration of rapid phenomena with unprecedented spatial resolution (< 100 μm) [24]. Concerning cardiac studies in rats, PETRUS uses a commercial pediatric/rheumatology ultrasound probe (SuperLinear™ SLH20-6, Supersonic Imagine, France; Fig. 2b) with negligible effects on PET image quality [9]. The probe is attached through a 35-cm-long hollow carbon rectangular cuboid (Polyplan Composites, France) to a six-degree-of-freedom high-precision micromotor (Hexapod H811, Physik Instrumente, Germany; minimum incremental motion 0.2 μm) fixed to the animal bed of the PET-CT scanner. A home-made 3D-printed plastic holder joins the carbon arm and the probe. Acoustic impedance coupling between the probe and the depilated skin of the animal is obtained using degassed ultrasound gel (Medi’gel Blue ECG, Drexco Medical). The automatic process of multimodal data co-registration between UUI and PET volumes (detailed in [23]) provides a mean accuracy of co-registration of 0.10 ± 0.03 mm. In the present study, we used UUI-B-mode, typically with a plane FOV of 25.6 mm × (20 to 30) mm, a pixel-size of 0.1 mm × 0.1 mm, and 16 temporal frames covering uniformly the full heart cycle.

To obtain the ultrasound B-mode images, we employ the research mode of our ultrafast scanner. We performed 500 frames-per-second (fps) acquisition employing flat research acquisition mode, spatial compounding using 5 beams orientation at − 6, − 3, 0, 3, and 6 degrees, and 15 MHz of central frequency. Spatial compounding is a classical method for speckle noise reduction that have demonstrated to avoid excessive speckle reduction. Besides, we combined this method with another classical image regularization aiming as well to improve image quality by noise speckle reduction consisting in post-filtering with Laplacian pyramid-based nonlinear diffusion, in which nonlinear diffusion filtering is applied to band-pass ultrasound images in using a multi-scale, Laplacian domain [41]. The total effect of the regularization employed is a set of images with reduced granularity and enhanced edges suitable for motion detection [42].

### Animal Experiments

Animal experiments were approved by the French Ethical Committee (approval number 18–146). We processed real data acquired with the PETRUS system from two successive imaging sessions of a 10-week-old female Wistar rat: (i) in normal conditions (baseline) and (ii) after surgically induced myocardial infarction. Baseline imaging was done 3 days before surgery. During surgery, the rat was under isoflurane anesthesia (2.5 %), and physiological parameters were constantly monitored. Analgesics were injected before and post-surgery. A permanent ligation of the left anterior descending coronary artery (LADCA) was performed after thoracotomy under endotracheal intubation. The incision was sutured, and the air in the thorax removed. The rat was imaged 4 h after LADCA ligation. For both imaging sessions, the rat was positioned on a customized bed (as in Fig. 2b) with ECG, temperature, and respiration monitoring. For the baseline and the infarcted cases, the mean registered animal’s heart rate was 260 and 277 beats per minute (bpm), respectively. We used a high level of isoflurane during imaging (~ 4 %) in order to limit the respiratory rate and minimize the effect of respiratory motion on PET acquisitions. The ultrasound probe was positioned over the chest to obtain a standard long-axis view of the myocardium with UUI-B-mode at a frame rate 500 fps for 5 s. UUI-B-mode acquisitions were triggered at the respiratory pause of the animal. A CT scan was acquired for attenuation correction of the PET data in semi-circular mode, with 50 kV, 720 projections, and 170 ms per projection. Attenuation correction was based on default settings of the PET-CT scanner using two tissues segmentation (tissue and air). Gated acquisition was started 30 min after the injection of 400 μL of ~ 41 MBq FDG in 0.9 % NaCl. The 30-min ECG-gated PET acquisition and UUI-B-mode were acquired simultaneously. From the sequence acquired with UUI-B-mode, a full cardiac cycle in respiratory pause was extracted using the simultaneously acquired ECG and respiratory signals. Next, the extracted frames were interpolated to 16 frames uniformly spaced in time. Every gated cardiac-PET image was reconstructed after removing heart cycles longer or shorter than 40 % of the mean nominal heart cycle duration along the whole experiment. PET data were reconstructed using the Tera-Tomo reconstruction engine (Mediso, Hungary) with 6 iterations and 4 subsets, scatter and attenuation correction, and with 16 temporal frames, covering the full cardiac cycle. Finally, the multimodal PETRUS data was co-registered. Long-axis view was chosen in order to explore the territory irrigated by the LADCA.

## Results

**Fig. 3:**
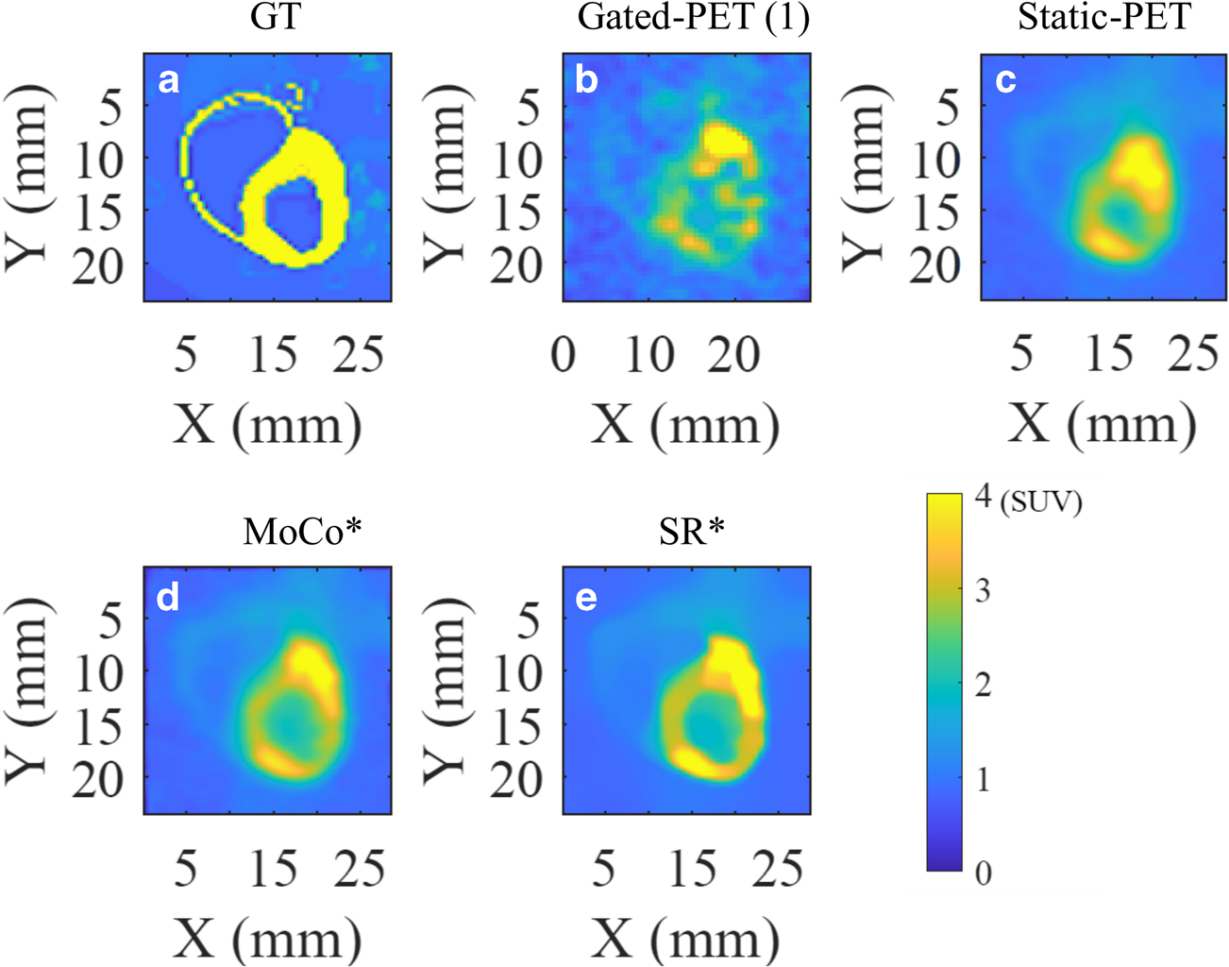
Numerical test using PET simulated data with the realistic rat phantom including both respiratory and cardiac modeling (ROBY) and the Monte Carlo software MCGPU-PET. **a** Ground-truth distribution of activity. **b** Gated PET image in end-diastole cardiac phase. **c** Static PET reconstruction. **d** Anatomy-based motion correction (MoCo). **e** Anatomy-based SR reconstruction. Asterisk (*) indicates that both images were obtained using as anatomic reference the real phantom activity distribution.

Regarding the simulated experiments, Fig. 3 summarizes the results of this test and shows the reference end-diastole cardiac phase, the gated-PET in end-diastole cardiac phase, the static-PET, and the anatomy-based SR image. Compared with the ground-truth image, image quality of the gated-PET and static-PET images is notably degraded. The definition of the structure of the wall is partially lost as well as the quantitative information. However, both the MoCo and SR image are qualitatively and quantitatively improved in that respect. Quantification of the selected ROIs are reported in Table 1.

**Table 1.** Quantitative analysis of treated images

	ROI	Gated #1	S-PET	MoCo	SR-PET
Numerical experiments	Wall (SUV)*	3.70 ± 0.24	3.53 ± 0.12	3.56 ± 0.09	3.92 ± 0.04
	Backg (SUV)**	1.36 ± 0.10	1.60 ± 0.07	1.58 ± 0.02	1.31 ± 0.01
	Contrast (ad)	1.73	1.21	1.26	2.00
	SNR (dB)	11.60	14.22	17.53	20.07
	S. Res. (mm)	1.79	1.89	1.64	0.84
Animal experiments					
BL	Wall	3.94 ± 0.22	3.63 ± 0.07	3.68 ± 0.07	4.27 ± 0.07
	Backg.	1.19 ± 0.10	1.33 ± 0.08	1.23 ± 0.06	1.01 ± 0.05
	Contrast (ad)	2.30	1.72	1.99	3.22
	SNR (dB)	11.60	14.57	15.09	15.58
	S. Res. (mm)	1.59	1.99	1.94	0.85
I	Wall	3.50 ± 0.29	2.80 ± 0.06	2.63 ± 0.06	3.88 ± 0.04
	Backg.	1.29 ± 0.09	1.37 ± 0.06	1.41 ± 0.03	1.15 ± 0.03
	Lesion	1.69 ± 0.14	1.56 ± 0.04	1.76 ± 0.04	2.18 ± 0.03
	Contrast (ad)	1.72	1.04	0.87	2.37
	SNR (dB)	11.17	15.35	16.46	17.55
	S. Res. (mm)	1.66	2.01	1.88	0.90

*I* infarcted, *BL* baseline, *S-PET* = static-PET, *MoCo* = motion corrected, *Gated #1=* first frame of gated-PET, *SR-PET =* Super-resolution PET

*Reference value SUV = 4

**Reference value SUV = 1

We observed that the deviations between the mean intensity values in the evaluated ROIs and their expected values were smaller in the ROIs located in the ventricle’s wall than the ones located inside the ventricle’s cavity (Table 1). This is expected because inside the cavity the PET signal is considerable smaller than in the tissue and because this region does not present tissue in motion that could be used to increase the spatial sampling.

In the ventricle’s wall, the smaller deviations between mean and expected values in the ROIs were obtained for the SR images (2 %), followed by the gated- PET (7 %), the MoCo (11 %), and the static-PET images (12 %). However, the image quality of the frames of the gated-PET was very low (see Fig. 3), preventing their diagnostic function and reliability. The deviations between expected and mean values were improved in a 7 % after applying MoCo and in an 82 % after applying SR in comparison with the static PET. Taking the MoCo images as reference, the SR treatment provided 81 % more reliable quantitative values than MoCo ones.

In terms of image quality parameters, using the static-PET images as reference, we observed an improvement of 4 % in terms of contrast, of 23 % in terms of SNR, and 13 % in terms of spatial resolution of the MoCo images, while in the SR image these values improved in 66 %, 41 %, and 55 % for the contrast, SNR, and spatial resolution, respectively. Taking the MoCo as reference, the SR images provides 60 %, 14 %, and 48 % better values of contrast, SNR, and spatial resolution, respectively.

**Fig. 4:**
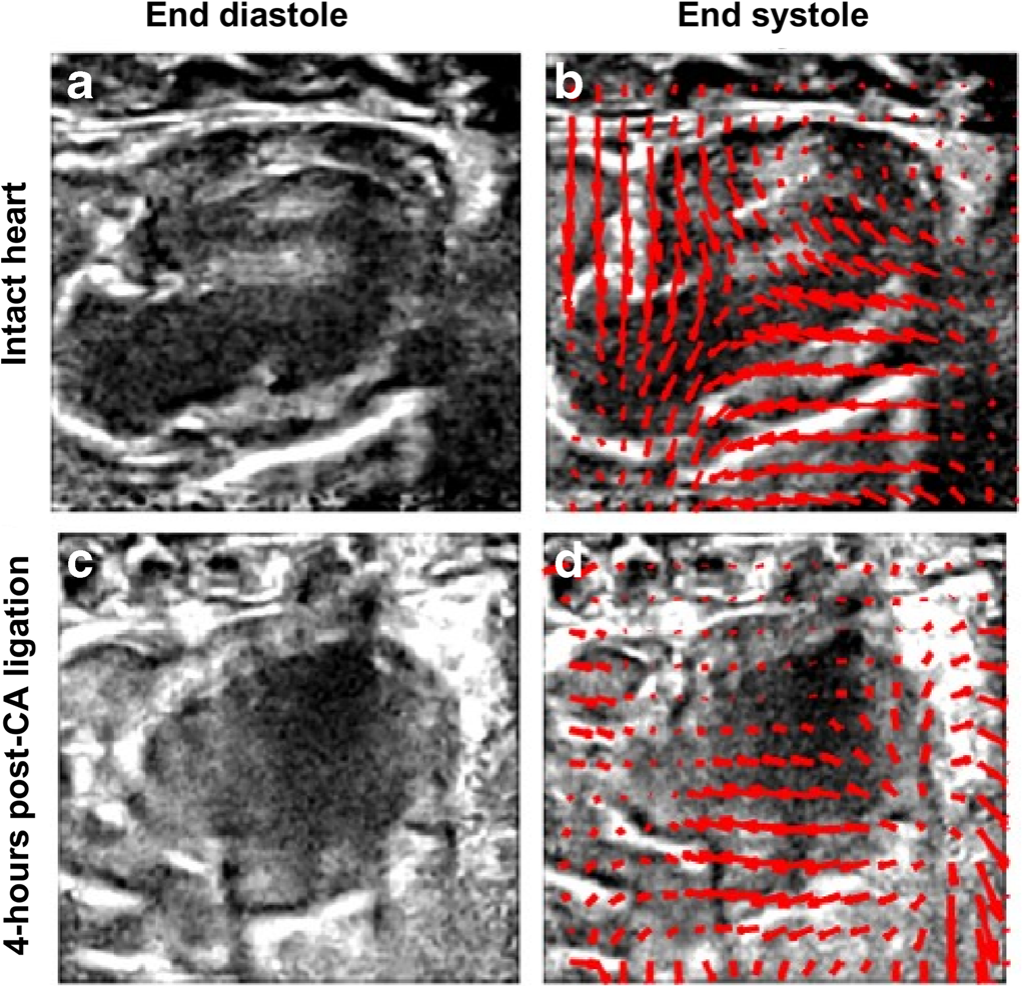
Fusion of B-mode ultrasound images and motion vector field (as red arrows) of the cardiac deformation from end-diastole (**a**, **c**) to end-systole phases (**b**, **d**). Panels **a** and **b** represent an intact heart and panels **c** and **d** 4-h after left-descending coronary artery ligation.

**Fig. 5:**
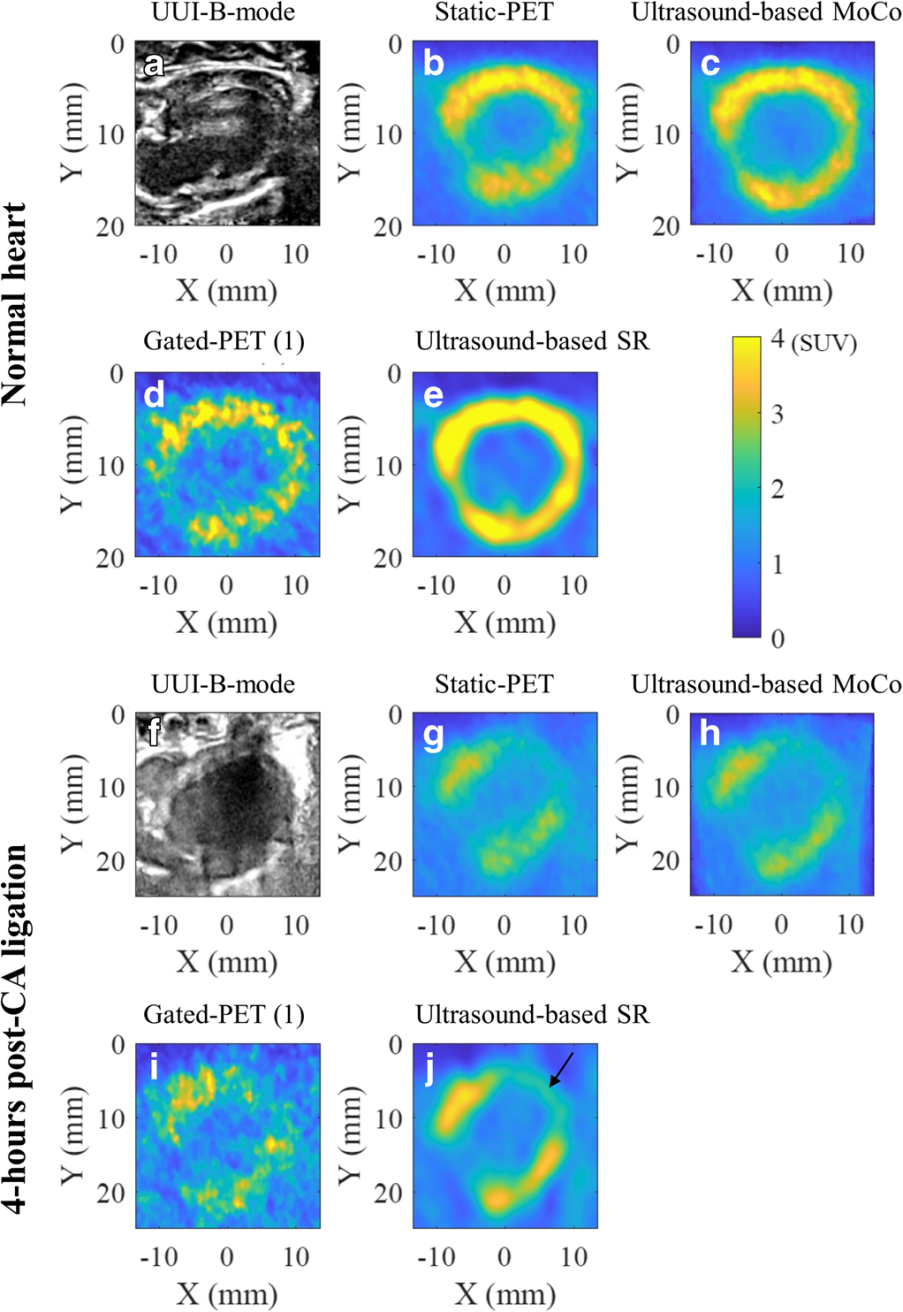
Multimodal PETRUS images of an intact (**a**–**e**) and 4-h post left-descending coronary artery ligation (**f–j**) rat heart. **a**, **f** UUI-B-mode image in long-axis orientation and end-diastole phase. **b**, **g** Static PET images. **c**, **h** Ultrasound-based MoCo images. **d**, **i** Gated PET in end-diastole frame. **e**, **j** Ultrasound-based SR images. The black arrow in **j** points towards the ischemic area.

Regarding the *in vivo* experiments, Fig. 4 shows the motion vector fields between diastole and systole cardiac phases for the intact and ischemic hearts. The overall motion seems to be correctly identified, and the mechanical affectation of the ischemic heart can be well appreciated. Figure 5 shows in end-diastole cardiac phase, UUI-B-mode, gated PET, MoCo, ultrasound-based SR, and static PET images, in intact and infarcted rat hearts. Table 1 reports the quantification performed over those images. Qualitatively, in the MoCo and ultrasound-based SR images, the FDG uptake sharply delineated the walls of the left ventricle. However, the ultrasound-based SR images present less spillover outside ventricle’s wall tissues. Additionally, the ultrasound-based SR image clearly identified the metabolic remodeling in the ischemic myocardium corresponding to the vascular territory of the ligated LADCA, evidencing a small but still active FDG uptake in the ischemic region that could not be properly appreciated in the rest of available images.

 Taking the static-PET images as reference, the mean values in the ROIs increased by 4 % in the intact ventricle’s wall and by 13 % in the ischemic region when MoCo was applied, and by 28 % and 40 % in the intact ventricle’s wall and in the ischemic region, respectively, when ultrasound-based SR was applied. Using as reference the MoCo images, the ultrasound-based SR provided 32 % and 40 % higher SUV values in the ventricle’s wall and in the ischemic region, respectively. Compared to those of the static images MoCo provided a modest improvement of the image quality parameters, 3 %, 5 %, and 5 % for the contrast, SNR, and spatial resolution, respectively. However, utrasound-based SR yielded 102 %, 11 %, and 56 % higher contrast, SNR, and spatial resolution, respectively, than static PET images. Comparing MoCo and ultrasound-based SR images, the latter provided 95 %, 5 %, and 54 % better contrast, SNR, and spatial resolution, respectively, than the former. Overall, ultrasound-based SR yielded submillimetric spatial resolution with an average value of 0.88 mm.

## Discussion

The previous results support the hypothesis that ultrasound-based SR improves the spatial resolution of the images without affecting the SNR, which is a basic precept of the technique. Analogously, all the image quality parameters evaluated were considerably improved, facilitating the identification of heart lesions, which appear enhanced in the images as a result of a better spatial resolution and decreased PVE. The total amount of counts present in the entire gated-PET sequence (2.35 × 10^10^ and 2.63 × 10^10^counts for the intact and infarcted hearts, respectively) were not substantially modified by the ultrasound-based SR algorithm (2.35×10^10^counts and 2.65 ×10^10^counts for the intact and infarcted heart). Importantly, the ultrasound image acquisition is performed during the PET acquisition and provides images in real time. Co-registration of multimodal data takes 2 min on a dual core Intel(R) Xeon(R) CPU E-5-2637 v4 @ 3.5GHz, and most of this time is used for data loading of the PET volumes. Ultrasound-based SR adds a short computation time to the reconstruction process. The motion registration of 16 UUI-B-mode frames takes 1 min, and the SR algorithm 1 min on the same machine. Both codes were run in non-parallelized conditions, while parallelization is likely to improve considerably the execution time.

In this study we used gated-PET reconstructions of 16 frames/cycle associated to 16 UUI-B-mode anatomical frames/cycle. The reconstruction time for a 30-min acquisition using 16 gates and the commercial software of MEDISO run on the dedicated workstation is ~  5.5 h, and ~ 10.4 h for 32 gates. The SNR from 16 to 32 gates, using as example the intact heart rat examination described in “Animal Experiments,” decreased in ~ 40 %, leading to a considerable loss of signal. Therefore, in the present configuration there is no advantage to explore much higher frame rates even if they are compatible with UUI, if onr is to keep reasonable reconstruction times and good PET image quality.

One limitation of the present study is that our PET-CT scanner did not allow to double trigger PET data acquisition. Hence, we used a high level of isoflurane (~ 4 %) in order to limit the respiratory rate and minimize the effect of respiratory motion on PET acquisitions. In these conditions, the observed cardiac displacement due to respiration estimated from the B-mode image was ~ 0.7 mm, which is smaller than our PET’s spatial resolution. Additionally, it is noteworthy that, in spite of the simplicity provided by in-image-domain SR, reconstruction-incorporated SR methods have also been reported as in [43] and may represent a future extension of the present study.

The SR method developed in this work uses 2D ultrasonic sequences, while the beating heart undergoes complex movements in 3D. However, the rapid progresses of 3D ultrasound probes, some of which are already in exploitation in clinical and preclinical practice, will allow 3D SR in the near future [44]. Moreover, the use of MVF in 3D obtained from ultrasound may indeed be a convenient methodology to create 4D virtual X-rays CTs to provide attenuation correction in PET images [22].

A particularity of this study is that it requires a temporal framing to sample the cardiac cycle superior to the one employed in classical echocardiography. Dedicated clinical echocardiography systems provide sequences allowing up to 50–80 fps, which is sufficient to image the resting heart beating (in humans is ~ 70 bpm) [45]. However, to perform stress echocardiography or preclinical imagining, such temporal framing results insufficient to sample the heart cycle [45]. Therefore, other imaging schemes to increase frame rate while keeping high image quality are needed, such as cardiac-UUI (the technique employed in this study) or multi-line transmit technique [24, 46]. In particular, UUI technology offers a large diversity of functional imaging information such as the measurement of cardiac contraction parameters and myocardial elasticity [23, 24], which could be used in combination with high-resolution metabolic imaging of the heart.

## Conclusions

We presented the proof-of-concept that ultrasound-based SR techniques based on co-registered, simultaneously acquired PET and ultrasonic data drastically enhance the quality of metabolic images of the rodent heart in terms of spatial resolution, SNR, and contrast. The performance of the method was demonstrated using numerical and animal data. In the infarcted rat heart, ultrasound-based SR improved the delineation of the metabolic defect due to the post-ischemic lesion. Ultrasound-based SR cardiac-PET allows precise imaging of cardiac metabolism in pathological situations characterized by cardiac glucose dysregulation.

## Electronic Supplementary Material

ESM 1 (DOCX 517 kb)
