# Supplementary material for: Ultrafast Ultrasound Imaging for Super-Resolution Preclinical Cardiac PET
Source: Mol Imaging Biol. 2020 Jun 29;22(5):1342–52. doi: 10.1007/s11307-020-01512-w (PMC7497458; doi:10.1007/s11307-020-01512-w)
Supplement: Supplementary file 1 — (DOCX 517 kb) [file 11307_2020_1512_MOESM1_ESM.docx]

**Supplementary material: Validation of the motion registration algorithm.**

To test the performance of our motion registration method, we used a numerical phantom (“Phantom A”) simulating a real B-mode acquisition of a rat heart in long axis view. We applied a controlled analytical deformation to a real end-diastole single frame UUI-B-mode. The analytical expression to describe the movement of each pixel was modified from (1) and adapted to the size, orientation and specificities of the motion of the rat heart:

$$\begin{aligned} M_{sim}=\sin^{1.5} \left( \frac{\pi t}{T} \right)\left[ \begin{matrix} A_{u}\cdot\left( \frac{y_{apex}-y}{y_{apex}-y_{valve}} \right) \\ A_{v}\cdot\left( \frac{x-x_{ox}}{x_{endo}-x_{ao}} \right) \end{matrix} \right],\#(SM-1) \end{aligned}$$

where $M^{sim}$ are the simulated reference displacements along the $x$ and $y$ axis of the plane, $T$ represents the number of frames,$y_{apex}$ is the position in the $y$-axis of the myocardial apex, $y_{valve}$ is the position in the $y$-axis of the mitral valve, $x_{ox}$ is the reference origin of motion in the $x$-axis, and $x_{endo}$ is the position in the $x$-axis of the endocardium. $A_{u}$ and $A_{v}$ are the amplitudes of movement in each direction of the plane. Fig. SM-1 represents the fusion of end-diastole and end-systole frames simulated with the previous model.

To analyze the accuracy of the motion registration method, the total root-mean-square-error (RMSE) between the registered template and reference images was calculated. Additionally, we calculated the mean pixel-wise discrepancy between the simulated and estimated displacement as:

$$\begin{aligned} discrepancies \left( \% \right)= \\ \frac{1}{2}\sum\left[ \left( \frac{\left( M_{sim}-M_{est} \right)}{M_{sim}} \right) \right]\cdot100,\#(SM-2) \end{aligned}$$

where $M_{est}$are the displacements estimated with our registration method. Additionally, we added 10 dB of Gaussian noise to the simulated images and degraded the intensity of (i) the reference image, and (ii) the reference and template images in a given sector. We used an exponentially increasing attenuation of the intensity from the top to the bottom of the image with 90% attenuation at the bottom of the sector (see Fig. SM-1 C and D).

Fig. SM-1, shows the results of a registration performed with the proposed method. This test allowed us to empirically select the parameter k=0.5 in Eq. 8 of the text, as this value provided the lower discrepancies between the simulated and estimated motion fields with a fixed number of iterations. Panels A and B show the images before and after registration, respectively. Panel C depicts the displacement vectors (plotted 1 every 20 pixels) simulated (in red, using Eq. SM-2) and estimated with our MoCo method (in green). The mean discrepancy between the two motion fields was 17 %, suggesting a correct agreement between both groups of displacement vectors. From a qualitative point of view, both the direction and magnitude of the displacement vectors present a high level of resemblance between both cases. In the presence of (i) added white noise and (ii) added noise and intensity degradation (Fig. SM-1 D and E), the mean discrepancy between simulated and estimated displacement vectors was in both cases the same as in the non-degraded case, i.e. 17%, demonstrating robustness against noise and intensity variations. This suggests that the registration scheme proposed is suitable for the registration of pre-clinical cardiac ultrasound images of our Ultrafast-Ultrasound scanner.


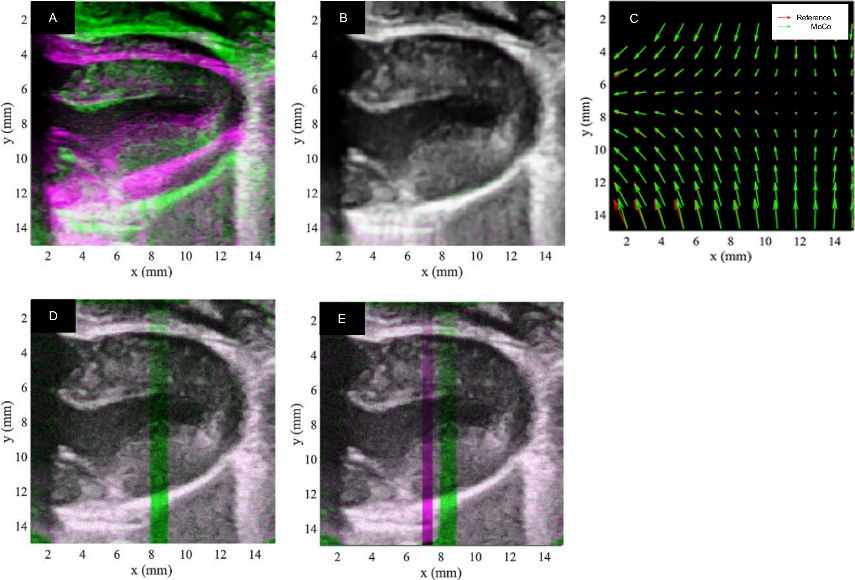


Fig. SM-1. A, overlay in two color bands of end-diastole (green) and end-systole frames (magenta), the gray regions represent pixels with similar intensities and magenta and green regions different ones. B, Overlay of frames after motion registration. C, Reference (red) and estimated (green) displacement vectors for simulated phantom A. Displacement vectors are represented every 20 pixels. D, like B but adding 10 dB of white noise to both image frames and an exponential degradation of intensity in a sector of the end-diastole frame. E, like C but degrading sectors of intensity in both end-diastole and end-systole frames.

References

1. Ledesma-Carbayo MJ, Kybic J, Desco M, Santos A, Suhling M, Hunziker P, et al. Spatio-temporal nonrigid registration for ultrasound cardiac motion estimation. IEEE Trans Med Imaging. 2005;24(9):1113-26.
